# The Development of Two High-Yield and High-Quality Functional Rice Cultivars Using Marker-Assisted Selection and Conventional Breeding Methods

**DOI:** 10.3390/ijms23094678

**Published:** 2022-04-23

**Authors:** Yong-Pei Wu, Yu-Chi Chang, Hsin-I Kuo, Bing-Nan Lin, Shu-Mei Wang, Yu-Chien Tseng

**Affiliations:** 1 Department of Agronomy, Chiayi Agricultural Experiment Station, Taiwan Agricultural Research Institute, Chiayi City 60044, Taiwan; wuypei@tari.gov.tw (Y.-P.W.); ycc71719@gmail.com (Y.-C.C.); 2 Department of Agronomy, National Chiayi University, Chiayi City 60004, Taiwan; s1070043@alumni.ncyu.edu.tw (H.-I.K.); qaz612197@gmail.com (B.-N.L.); 3 Department of Bio-Industry Communication and Development, National Taiwan University, Taipei 10617, Taiwan; wangsm@ntu.edu.tw

**Keywords:** functional food, biofortification, marker-assisted selection, giant embryo, aldehyde dehydrogenase, colored rice

## Abstract

Rice (*Oryza sativa* L.) is an important crop worldwide. Functional rice has exhibited health benefits. The aim of this study was to use marker-assisted selection (MAS) to introgress two genes, *GE* (giant embryo) and *OsALDH7* (aldehyde dehydrogenase, golden-like endosperm) into colored rice and obtain high yield functional rice. CNY103108 and CNY103107 are two rice lines with golden-like endosperms and giant embryos. They were used as the donor parents. CNY922401, an elite purple waxy rice line, and TNGSW26, an indica red waxy rice cultivar were used as the recurrent parents. Foreground selection of the progenies was completed using functional markers for *GE* and *OsALDH7*, and background selection was completed using molecular markers to recover the background of the recurrent parents. MAS results showed a purple functional rice population (PFR) (CNY922401/CNY103108), with the recovery rate of the recurrent parental genome as 91.3%, and a red functional rice population (RFR) (TNGSW26/CNY103107) with the recovery as 89.8%. After five-season yield trials and several antioxidant activities analyses, PFR32 and RFR13 lines, which have similar yields and antioxidant activities, were selected as the recurrent parents with a golden-like endosperm and a giant embryo. For a biofortification purpose, they can become valuable products and be adapted to the current agricultural community.

## 1. Introduction

Rice (*Oryza sativa*) is an economically important agronomic crop. It is the second most produced crop in the world (FAOSTAT, https://www.fao.org/faostat/en/#data (accessed on 13 March 2022) and more than 50% of the world’s population consumes rice as the main daily food. In developed countries, due to changes in dietary habits, consumption of gourmet food has become popular, which has led to health issues such as high blood sugar (hyperglycemia), high blood pressure (hypertension), hyperlipidemia, and diabetes. On the other hand, in areas with continuous food shortages, people suffer from severe nutritional deficiencies. Malnutrition is responsible for more than half a million deaths in the world [1]. In order to overcome these problems, the rice breeding program of the International Rice Research Institute (IRRI) has been working on improving rice quality and increasing its nutritional and functional values [2].

“Functional rice”, is defined as rice with functional active ingredients in the endosperm, embryo, and bran that can improve the human metabolism, and not only provide energy, but also fulfill nutritional needs and prevent disease. Rice contains many functional active ingredients including iron, zinc, γ-aminobutyric acid (GABA), inositol hexaphosphate (IP-6), ferulic acid, tocopherol, tocotrienols, inositol, and vitamins [3]. These active ingredients of functional and nutritional rice affect the metabolism in humans, leading to health benefits. Biofortification is a process that increases the nutrient content of food crops through conventional plant breeding without sacrificing any characteristics that are preferred by consumers or farmers [4]. In Japan, the development of fortified foods has become an important issue, and several products have been produced, such as *Coenzyme-Q10* (CoQ10)-enriched rice [5] and high-lysine rice [6]. Functional rice improves human health by providing antioxidants and lowering cholesterol and blood sugar levels [7,8,9]. Friedman [10] reported that the active ingredients in rice bran and hull exert numerous health-promoting effects (antioxidative, anti-inflammatory, and immunostimulatory effects) on animals and humans.

Besides rice, blue maize has nutraceutical properties with higher flavonoid and anthocyanin [11,12]. The blue maize hybrid has been made and contains high levels of nutraceuticals similar to blue maize landrace with excellent potential for commercial planting and industrial utilization [13]. Purple potato, purple sweet potato, and black soybean are other functional food examples. Han [14] reported that the anthocyanin-rich purple potato flake extract has an antioxidant capacity and improves the antioxidant potential in rats. Purple sweet potato is rich in anthocyanin and can improve the human diet. Thirty purple sweet potato varieties were analyzed quantitatively and qualitatively, and seven identified anthocyanins were considered as key constituents of the bioactivity [15]. Black soybeans are beneficial to humans because of the healthy components such as isoflavones, anthocyanins, and saponins. In particular, isoflavones are well known to reduce cholesterol risk [16,17].

Giant embryo rice is a type of functional rice that has a larger embryo than normal rice. GABA levels increase and accumulate rapidly in giant embryo rice after the rice is soaked in water. GABA is an amino acid and a neurotransmitter that can slow activity in the brain, producing a calming effect. Research has shown that patients fed GABA-rich rice had improved hypertension compared to that of the placebo group [18]. Several giant embryo rice mutants have been reported to be controlled by the same gene, the *GIANT EMBRYO* gene (*GE*), but with different alleles [19,20]. *GE* controls the size balance between the embryo and the endosperm. It encodes the CYP78A13 protein, which is related to cytochrome P450, and at least ten alleles have been reported for this gene [21].

Golden rice is a transgenic rice, which was created to prevent vitamin A deficiency in some developing countries [22]; in golden rice, phytoene accumulates in the endosperm, which introduces the ability to produce β-carotene [23], and the genes responsible for this characteristic have been introgressed into different lines [24]. The Taiwan Agricultural Research Institute, Chiayi Agricultural Experiment Station (TARI, CAES) developed the cultivar Tainung76 (TNG76) using mutagen sodium azide. TNG 76 is light yellow in color, and the special color was due to a mutation in the *aldehyde dehydrogenase* (*aldh7*) gene (LOC_Os09g26880) on chromosome 9. A single nucleotide mutation (A to G) happened on exon 11 (position: 16333837) and caused the amino acid arginine to change to glycine, which caused a metabolic pathway difference in the endosperm and resulted in the accumulation of a yellow substance [25,26]. Compared to Tainung 67 (TNG 67), the original cultivar used for mutagenesis, TNG 76 has a higher flavonoid and vitamin E accumulation [27] (Table S1).

Colored rice contains higher anthocyanin levels, which lead to purple-, red-, and black-colored grains. Several studies on colored rice have been conducted [28,29]. Colored rice provides a higher nutrient content, such as minerals, vitamin B1, iron, zinc, manganese, and phosphorus [30,31]. The antioxidant activity and total phenolic content of colored rice are also higher than those of white rice [32,33].

At present, marker-assisted selection (MAS) is an important tool for rice plant breeding [34]. The development of functional rice can also be conducted using MAS. Since the Giant embryo gene (*GE*) and golden-like rice gene (*OsALDH7*) have been identified, the molecular markers linked to these genes and the functional markers derived from these genes can be designed for breeding programs. The TARI, CAES has made efforts to develop different functional rice cultivars, including the giant embryo rice (Tainung78, TNG78), golden-like rice (Tainung76, TNG76), purple waxy rice (CNY922401), and red waxy rice (TNGSW 26). All cultivars exhibited a high yield and high quality with respect to their corresponding functional rice characteristics. The objectives of this study were to introgress giant embryo and golden-like rice genes into colored rice (purple and red) using MAS and through conventional breeding methods to develop two high-yield and high-quality functional rice cultivars.

## 2. Results

### 2.1. Base Population

**Figure 1:**
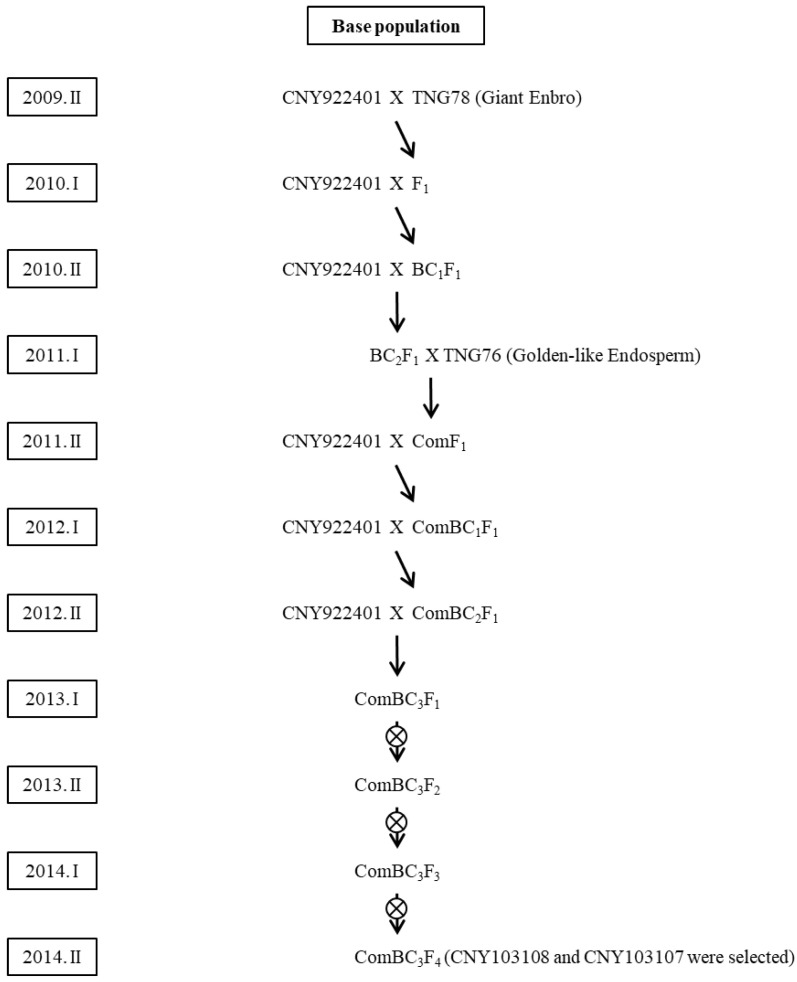
Breeding scheme for the base population.

The base population was derived from a backcross between a recurrent parent and two donor parents (Figure 1). The first donor parent, TNG78 (giant embryo), was crossed with CNY922401 (recurrent parent) to produce the F_1_ progeny. CNY922401 is a waxy rice breeding line with a purple color; it contains high anthocyanin and mineral contents, and a high yield. TNG78 is a *japonica* cultivar generated by the mutagen N-methyl-N nitrosourea (NMU). Its embryo is three times bigger than that of normal rice. Fine mapping revealed that this giant embryo gene of the base population was similar to *GE*, which is located on chromosome 7 [35,36]. MAS was used for both foreground and background selection to maintain the *GE* gene and the CNY922401 background.

After backcrossing with CNY922401 twice (backcross with F_1_ and BC_1_F_1_), the BC_2_F_1_ generation was obtained, which was crossed with the second donor parent TNG 76 (Golden-like endosperm) (Figure 1). TNG76 is a *japonica* cultivar generated from a mutant Tainung67 (TNG67) population by the mutagen sodium azide. It has a light-yellow appearance, which is very similar to that of golden rice. Sequencing results revealed that the sequence of its golden-like rice gene was similar to that of *OsALDH*7, which is located on chromosome 9 [25,26]. The ComF_1_ progeny was generated from the BC_2_F_1_ and TNG76 lines. ComF_1_ was continuously backcrossed with CNY922401 to obtain the ComBC_3_F_2_ generation. MAS was conducted for foreground (*GE* and *OsALDH*7) and background selection (CNY922401). Two breeding lines (CNY103107 and CNY103108) were selected from the ComBC_3_F_2_ generation, and both lines had yellow endosperm and giant embryo traits; however, they did not show a satisfactory yield and quality compared to that of their recurrent parent (CNY922401). Therefore, these two lines were used as parents to create two other populations—the purple functional rice population (PFR) and the red functional rice population (RFR).

### 2.2. CNY922401/CNY103018 (PFR Population)

**Figure 2:**
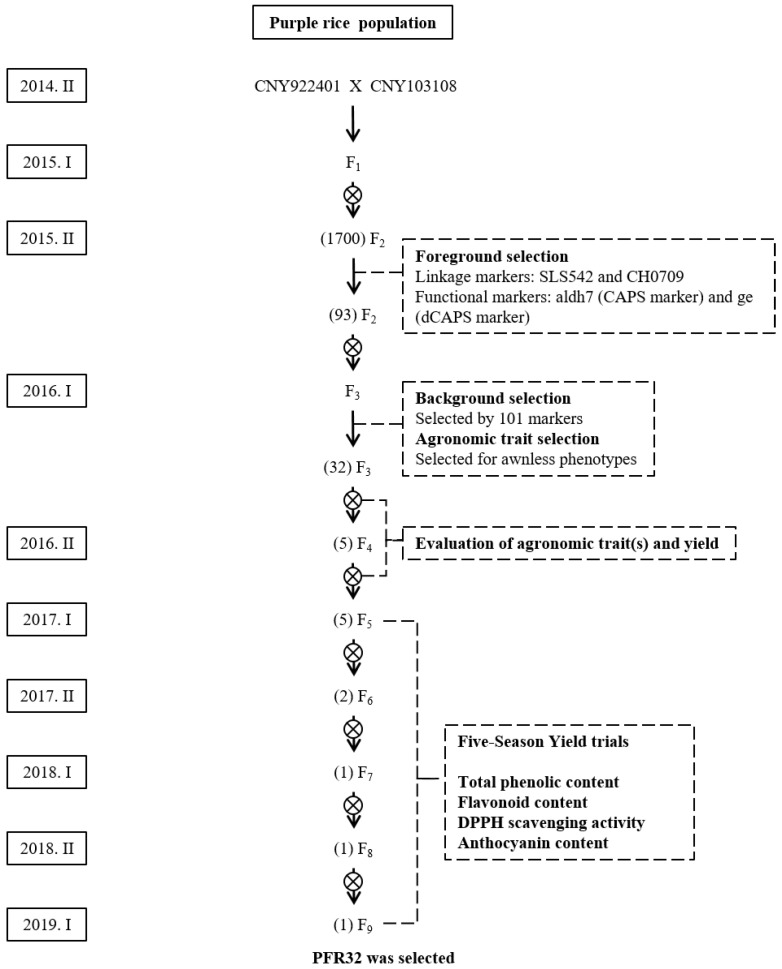
The breeding scheme for the purple functional rice population (PFR). Molecular markers were used for both foreground and background selections. Yield trials and antioxidant activity evaluations were performed. The numbers of the selected lines are enclosed in parentheses.

**Figure 3:**
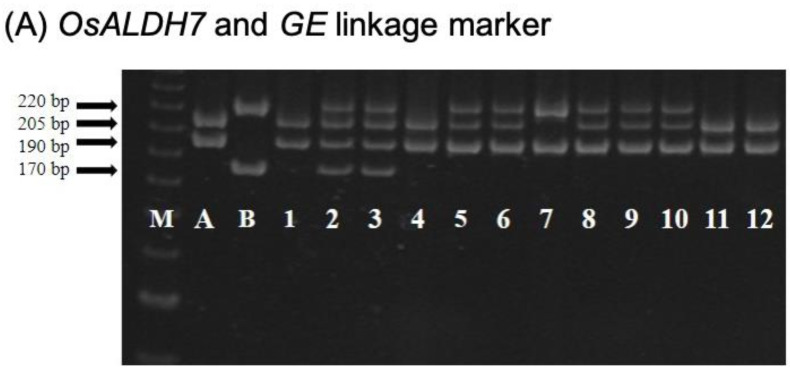

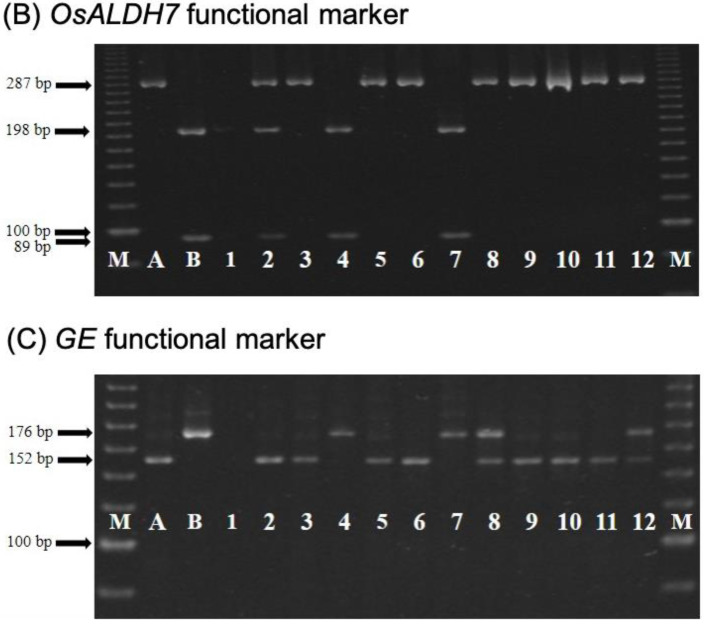
Foreground selection results for different genotypes using linkage and functional markers. (**A**) Genotype selection results using *OsALDH7* and *GE* linkage markers. (**B**) Genotype selection results using *OsALDH7* functional markers. (**C**) Genotype selection results using *GE* functional markers. M indicates a 100 bp ladder. A indicates the donor parents (CNY103108 and CNY103107). B indicates the recurrent parent (CNY922401 and TNGSW26). Numbers 1–12 indicate different breeding lines.

After crossing CNY922401 and CNY103108, approximately 100 F_1_ plants were generated and planted in the field (Figure 2). The true F_1_ hybrid plants were selfed to produce F_2_ seeds. The F_2_ seeds were milled and checked for their appearance on rice hulls before being planted, and only plants with a larger embryo and a purple seed color were selected; 1700 F_2_ seeds were chosen and germinated in hydroponic trays. Foreground selection was performed using SLS542 and CH0709 markers, which are tightly linked to the *OsALDH7* and *GE*, respectively. The analysis of the SLS542 marker revealed that if the individuals were linked to *OsALDH7*, a 205 base pair (bp) band pattern was observed, which was derived from the donor parent CNY103108. If the individuals were not linked to *OsALDH7*, a 220 bp band pattern was observed, which was derived from the recurrent parent CNY922401 (Figure 3A). The CH0709 marker was used to select the individuals tightly linked to *GE*, and the F_2_ plants linked to *GE* showed a 190 bp band pattern, similar to that of the donor parent CNY103108. The individuals with a 170 bp band pattern indicated that their genotypes were similar to those of the recurrent parent CNY922401 (Figure 3A).

**Figure 4:**
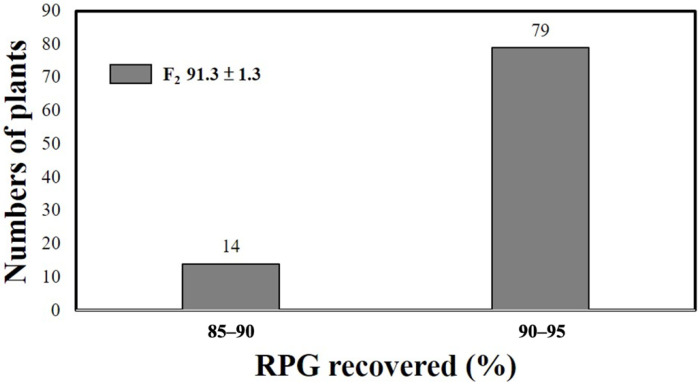
Frequency distribution of the recurrent parent genome (RPG) recovery rate estimated by markers in the F_2_ generation of the purple functional rice population.

Seeds that had both a 205 bp pattern (*OsALDH7*) for the SLS542 marker and a 190 bp pattern (*GE*) for the CH0709 marker were selected and screened for the functional markers aldh7 (Cleaved Amplified Polymorphic Sequence (CAPS) marker) and ge (Derived Cleaved Amplified Polymorphic Sequence (dCAPS) marker). In the case of the aldh7 functional marker, seeds with *OsALDH7* had a 287 bp amplicon (Figure 3B). The plants with the *GE* showed a 152 bp amplicon when screened for the ge functional marker (Figure 3C). After the two-stage foreground selection, 93 F_2_ plants were selected (Figure 2). These plants were then transplanted to the field, and 101 molecular markers, including 65 Simple Sequence Repeat (SSR) markers and 36 InDel markers were used for background selection to select individuals similar to CNY922401 (Figure S1, Table S2). CNY103108 was derived from CNY922401 but CNY103108 showed a poor germination rate and agronomic traits. The goal of the F_2_ generation was not only to maintain both golden-like rice and giant embryo characteristics derived from CNY103108, but also to obtain a genome background close to that of CNY922401. The genomic composition of each F_2_ plant was estimated using 101 markers. The recovery rates (frequency distribution) of the recurrent parent genome (RPG) are shown in Figure 4. The RPG of the F_2_ plants was similar to that of CNY922401, and the average RPG recovery rate was 91.3%.

**Figure 5:**
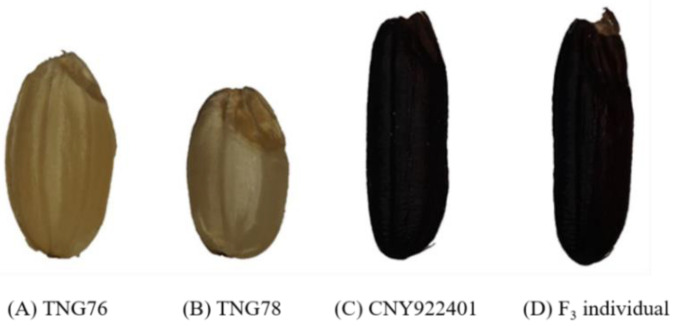
The grain appearance of brown rice for (**A**) TNG76, (**B**) TNG78, (**C**) CNY922401, and (**D**) F_3_ lines of the purple functional rice population.

In addition to the molecular markers, traditional agronomic performance was also considered (Figure 2), and 32 plants were selected, which became the F_3_ generation. The F_3_ generation was planted in a field with three replicates each. Each replicate contained 100 plants, and the yield was estimated after harvest. Five plants from five different F_3_ lines were selected based on their yield and pericarp color. Figure 5 shows the seed colors of TNG76, TNG78, CNY922401, and one of the selected F_3_ individuals. Only the plants with a purple pericarp and a high yield were selected and continued into the next generation. Five lines (PFR4, PFR5, PFR20, PFR24, and PFR32) were subjected to five seasonal yield trials (Table 1). The parental line (CNY922401) was listed as check, and yield trails were conducted with the RFR population, which was named as “RFR” with check (TNGSW26). The 2,2-diphenyl-1-picrylhydrazyl (DPPH) scavenging activity, and anthocyanin pigment content were calculated for both the PFR and RFR populations (Table 2). The five selected lines showed similar DPPH scavenging activity. All of the selected lines had a better anthocyanin pigment content than CNY922401, indicating that both the yield and quality of the selected lines were satisfactory.

After the first season yield trials (2017-1), three lines were eliminated, and two lines were retained (PFR5 and PFR32) (Figure 2). After the second season yield trial (2017-2), only the PFR32 line was retained. The yield performance of the PFR32 line for the following three seasons (2018-1, 2018-2, 2019-1) was similar to that of CNY922401 (Table 1). The total phenolic content, flavonoid content, DPPH scavenging activity, and anthocyanin pigment content of the CNY922401 and PFR32 lines in 2018 were similar (Table 3). The PFR32 line maintained the characteristics (waxy purple color) of its parental line (CNY922401), which included a high anthocyanin and mineral content, and a high yield. In addition, the PFR32 line possessed two genes, *GE* and *OsALDH7*, which are responsible for the giant embryo and golden-like rice characteristics, respectively. Compared to Taiwan’s popular high-quality cultivar Taiken 9, the PFR32 line showed a better DPPH scavenging activity and flavonoid and anthocyanin pigment contents, which enforces the importance of functional rice (Table 4).

### 2.3. TNGSW26/CNY103017 (RFR Population)

**Figure 6:**
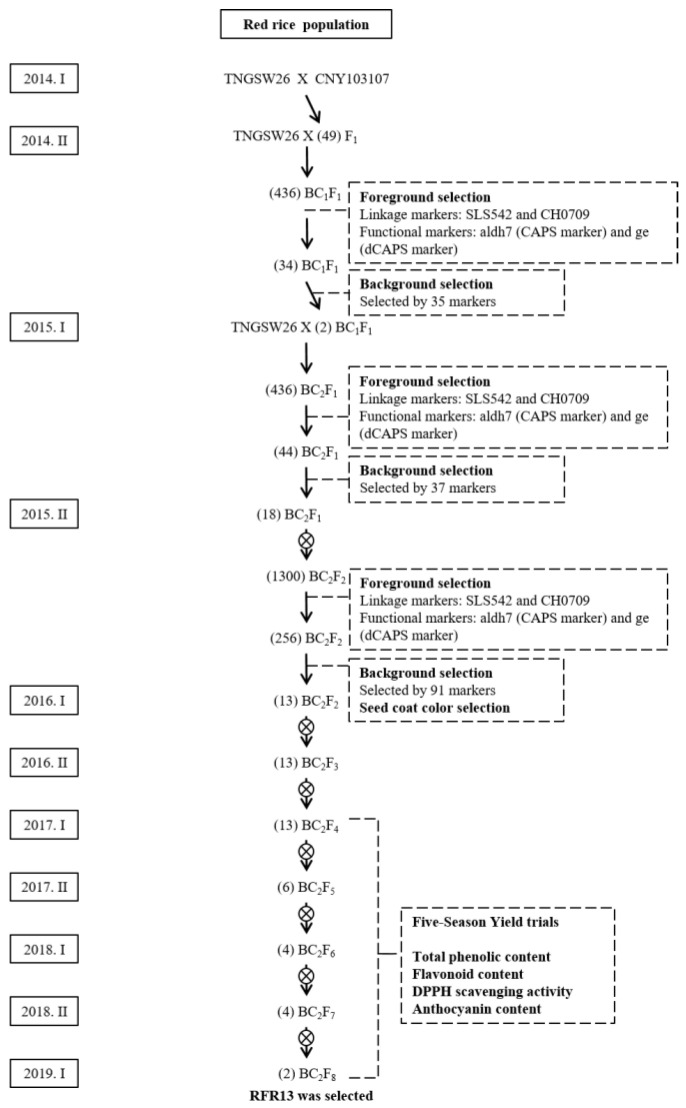
Breeding scheme for red functional rice populations (RFR). Molecular markers were used for both foreground and background selections. Yield trials and antioxidant activity evaluations were performed. The numbers of the selected lines are enclosed in parentheses.

**Figure 7:**
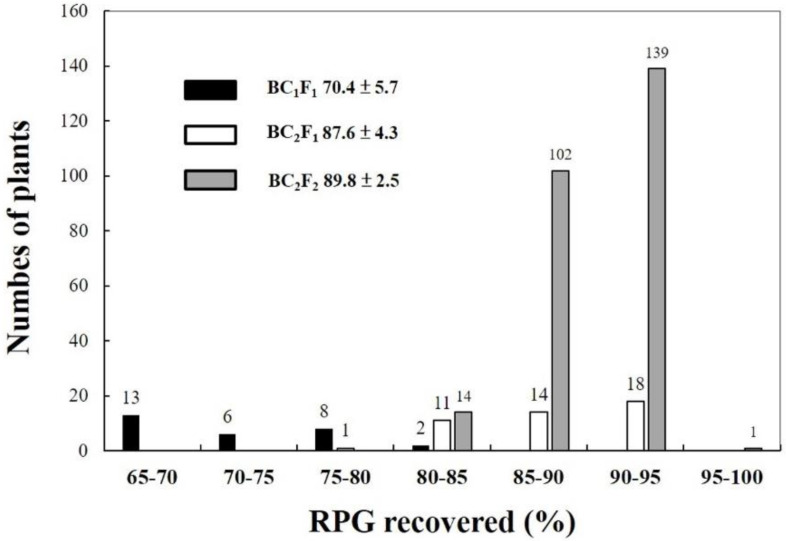
Frequency distribution of the recurrent parent genome (RPG) recovery rate estimated by markers in the BC_1_F_1_, BC_2_F_1_, and BC_2_F_2_ generations of the red functional rice population.

The RFR population is a backcross population derived from Tainung Sen Waxy 26 (TNGSW26)/CNY103107 (Figure 6). TNGSW26 is an elite cultivar of red waxy rice with a good yield and quality. It is resistant to rice blast, smaller brown planthopper, and white-backed planthopper. A total of 49 F_1_ plants were generated by hybridization. The F_1_ plants were backcrossed with the recurrent parent TNGSW26, to produce 436 BC_1_F_1_ plants. Similar to the process used for the PFR population, the foreground selection for the BC_1_F_1_ population was conducted using a two-step process during the seeding stage. The first step involved the use of SLS542 and CH0709 markers, which were tightly linked to the *OsALDH7* and *GE* genes, respectively (Figure 3A). Next, the heterozygous individuals (*OsALDH7/Osaldh7*, *GE/ge*) were screened with two functional markers: aldh7, a CAPS marker, and ge, a dCAPS marker (Figure 3B,C). After foreground selection, 34 plants showed heterozygous patterns and were transplanted into the field. Background selection was conducted on the BC_1_F_1_ population with 35 markers evenly distributed on 12 chromosomes (Figure S2, Table S3), including 28 SSR markers, 2 Sequence-Tagged Site (STS) markers, and 5 InDel markers. The average RPG recovery rate of the BC_1_F_1_ generation was 70.4%, ranging between 65% and 85% (Figure 7). A total of 2 plants, selected from the 34 plants of this generation, showed the best RPG recovery rate.

These two plants were backcrossed with TNGSW26 to produce 436 BC_2_F_1_ plants (Figure 6). Foreground selection was conducted using the procedure used for the BC_1_F_1_ generation; 44 plants were selected with both *GE/ge* and *OsALDH7/Osaldh7* heterozygosity. For background selection, 37 molecular markers were used, including 25 SSR, 5 STS, and 7 InDel markers (Figure S2, Table S3). These markers were different from the markers used for the BC_1_F_1_ population. Different marker sets that can avoid the same genome segments from the recurrent parent were selected repeatedly. The RPG recovery rate in the BC_2_F_1_ generation was between 79% and 95%, with an average of 87.6% (Figure 7). The 18 plants selected from the BC_2_F_1_ generation were selfed, and the harvested seeds were milled. Only seeds with a giant embryo and seed color close to that of TNGSW26 were retained. We obtained 1300 individuals in the BC_2_F_2_ generation (Figure 6). The same foreground selection procedure in BC_1_F_1_ and BC_2_F_1_ was performed at the seeding stage, and 256 plants were selected, which were planted in the field, and background selection was continued using 91 markers, including 69 SSR, 7 STS, and 15 indel markers (Figure S2). The RPG recovery rate for the BC_2_F_2_ generation was between 82% and 96%, with an average of 89.8% (Figure 7). Based on the RPG recovery rate and agronomic traits, 51 plants were selected and individually harvested to produce BC_2_F_3_ seeds.

**Figure 8:**
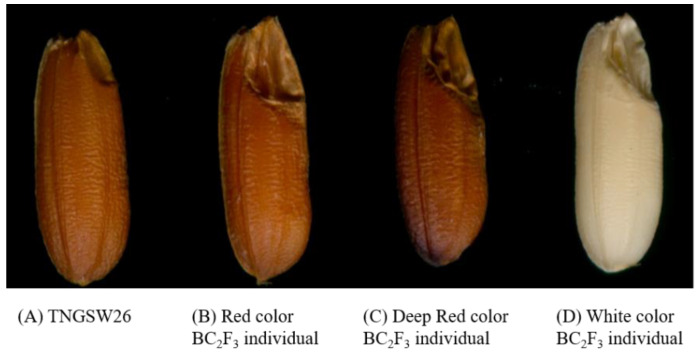
Grain appearance of brown rice for (**A**) TNGSW26 and (**B**–**D**) BC_2_F_3_ individuals, which had larger embryos than TNGSW26.

Based on the seed color of the BC_2_F_3_ seeds, they were classified into five groups as follows: 13 individuals in the red group, which was the same color as the recurrent parent TNGSW26; 4 individuals in the light red group; 15 in the dark group, 16 in the white group; and 3 in the mixed color group (white and red mixture). Most of the BC_2_F_3_ seeds, regardless of the color, had larger embryos than the recurrent parent TNGSW26 (Figure 8), and the giant embryo was the desired trait derived from the donor parent CNY103107. Only 13 individuals (RFR1, 2, 13, 16, 21, 22, 34, 36, 39, 44, 46, 48, and 51) from the red color group were retained for the five-season yield trial (Table 1). The trials were combined with the PFR population and checks (TNGSW26 and CNY922401). The DPPH scavenging activity was also calculated for these 13 lines (Table 2), and the results were considered as important selection guidelines.

After the first season yield trials (2017-1), seven lines were eliminated and six lines (RFR2, 13, 16, 36, 44, and 51) were retained for the next season trials (2017-2). In the yield trails of the next two seasons (2018-1 and 2018-2), only RFR13, RFR36, RFR44, and RFR51 were included (Table 1). As per the antioxidant activity evaluation in 2018, the phenolic content, flavonoid content, DPPH scavenging activity, and anthocyanin pigment content of the four selected lines were the same as those of the recurrent parent TNGSW 26 (Table 3). The phenolic content of RFR13 and RFR51 was better than that of TNGSW 26. After 2018 trials, two lines (RFR13 and RFR51) were retained; however, RFR51 was eliminated due to the poor yield performance in the 2019 yield trial (Table 1). Based on five season yield trials and two antioxidant trials (Table 1, Table 2 and Table 3), the yield performance of RFR13 was found to be similar to that of the recurrent parent (TNGSW26), which is an elite red waxy rice cultivar with a good yield and quality, and is resistant to rice blast, smaller brown planthopper, and white backed planthopper. RFR13 also possesses *GE* and *OsALDH*7, which are responsible for the giant embryo and golden-like rice characteristics, respectively. RFR13 had a higher flavonoid content and a better DPPH scavenging activity than TK 9, indicating the value of RFR13 as a functional food (Table 4).

## 3. Discussion

Conventional plant breeding focuses on direct phenotype selection, and MAS is an indirect selection method; however, environmental parameters can affect direct phenotype selection, making it difficult. MAS can be a useful tool for selecting low heritability traits, difficult or expensive measured traits, and developmental traits with late expression. More importantly, codominant markers can efficiently select against recessive alleles, which is difficult based on visual phenotype selection because heterozygotes can hide the recessive alleles and retain the allele frequency. Plant breeders commonly use the progeny test to remove heterozygotes from traditional backcrossing; however, this leads to the addition of another generation and increases the breeding time [37]. The strategies used to obtain next generation crops can be via conventional breeding or genetic engineering. The functional food created by conventional breeding is more acceptable to consumers than that by transgenic plants. The products from conventional breeding can also avoid the regulation that GMO (gene-modified organism) products should follow [38]. Several studies on vegetable crops have been conducted by zooming in on genes underlying QTL or by identifying these genes directly using genome-wide association studies (GWAS) [39,40]. MAS can become an excellent tool to produce functional food and achieve the biofortification goal without using the genetic modification technique.

In this study, backcrossing and MAS were combined to create marker-assisted backcrossing (MAB). For foreground selection (Figure 3), linkage markers (SLS542 and CH0709) and functional markers (aldh7 and ge) were used. Linkage markers are easier to deploy without complicated experimental procedures. However, the selection accuracy of recombinant individuals decreases. Functional marker usage involves restriction enzymes, and the cost is much higher with more complicated procedures, but it can accurately select the target genes and ensure that the genes of choice, i.e., *GE* and *OsALDH7* in this case, are incorporated. The new functional traits can be generated and kept through molecular markers. Background selection is another critical process in MAB. The RPG recovery rates were calculated using molecular markers (Figure 4 and Figure 7) that provide a way to realistically estimate the recurrent parent percentage rather than just the theoretical ratio. In this study, the RPG results confirmed the successful background selection using MAB. This is another marker benefit and made the conventional breeding program more efficient.

Quantitative trait loci (QTL) controlling embryo length and width were identified. For embryo length, there were three QTL located on chromosomes 1, 2, and 3, which showed 17.9%, 25.7%, and 9.2% PVE (phenotypic variation explained), respectively [41]. For embryo width, there were three QTL located on chromosomes 2, 8, and 10, which showed 13.5%, 15.7%, and 15.0% PVE, respectively [41]. In this study, the relative size ratio between the embryo and the endosperm was more critical. The giant embryo gene is mainly expressed on the margins of the embryo and endosperm [21]. Embryos were negatively regulated, which means that the *ge* mutant had a larger embryo and a smaller endosperm. However, the overexpression of *GE* can result in smaller embryos and larger endosperms [21]. Both PFR32 and RFR13 showed a giant embryo appearance by MAS and the trait shows the potential to produce the GABA-rich “germinated rice brown rice”.

In this study, the golden-like rice gene from TNG 76 was developed by an *ALDEHYDE DEHYDROGENASE* (*ALDH*) mutation [25,26]. Compared to Tainung 67 (TNG 67), the original cultivar used for mutagenesis, TNG 76 has a higher flavonoid and vitamin E accumulation [27] (Table S1). The DPPH assay is a method to assess the activity of antioxidants [42], and the DPPH assay revealed that TNG76 had a better free radical scavenger function than TNG67 [27] (Table S1). Shin et al. [43] used a T-DNA insertional mutant of *OsALDH7*, and the results indicated that *OsALDH7* is involved in removing various aldehydes formed by oxidative stress during seed desiccation. Mutation in *OsALDH7* impacts the ability of the mutant plant to withstand drought, salt conditions, and post-harvest storage [43]; however, in our study, we did not observe the malfunctions under the circumstances.

Zhang et al. [33] crossed normal white and purple rice and evaluated the total phenolic content and antioxidant ability of the progenies. The results showed that progenies with dark pericarps had a higher total phenolic content and a better anti-oxidation ability. In Sri Lanka, eight traditional, red-grained rice varieties were investigated, and their total antioxidant capacity and phenolic content were found to be seven times higher than those of the normal varieties. In addition, proanthocyanins have only been detected in traditional red varieties [32]. In this study, CNY922401 and TNGSW 26 were treated as recurrent parents and the progeny showed obvious purple and red colors (Figure 5 and Figure 8), which indicated the potential for a better antioxidant capacity and a higher phenolic content.

Both foreground and background selection were conducted, and the giant embryo and golden-like rice genes were successfully introgressed into colored rice (purple and red). Functional foods have great potential for the market; however, their yield is always an important issue in rice production. Rice cultivation in Taiwan involves a two-season cropping system, and the yield can vary between seasons; however, PFR32 and RFR13 continuously showed a promising yield performance over five seasons. Biofortified crops often cause yield penalties and increase difficulty in promoting the product [4]. PFR32 and RFR13 overcame the yield challenge and retained functional rice characteristics, such as high phenolic content, flavonoid content, DPPH scavenging activity, and anthocyanin content.

## 4. Materials and Methods

### 4.1. Plant Material

For the development of the base population (Figure 1), donor parents TNG78 (giant embryo) and TNG 76 (Golden-like endosperm) were included for backcrossing. The recurrent parent was CNY922401, which was waxy purple color rice, with high anthocyanin and mineral content, and a high yield. CNY103107 and CNY103108 lines were created in the base population; however, their yield and quality could be improved. Therefore, for PFR, CNY922401 was crossed with CNY103108 (Figure 2), and for RFR, TNGSW26, an elite red waxy rice cultivar, was crossed with CNY103107 (Figure 6).

### 4.2. Molecular Marker Design and Selection

Tightly linked markers (linkage markers) and functional markers were designed for foreground selection. Based on the sequencing data of *GE* and *OsALDH7*, the regions tightly linked to the mutation sites were selected. Primer SLS542 (forward: 5′-TCAAGAATATAAGCATTCGGA-3′, reverse: 5′-TACTCCCTCTGTTCAACGATA-3′) was tightly linked to the golden-like rice gene (*OsALDH7*), and primer CH0709 (forward: 5′-CACCATTAGAAGGCCAATAG-3′, reverse: 5′-ATCTTTTGCATGTCCTTACG-3′) was tightly linked to the *GIANT EMBRYO* gene (*GE*) (Figure 3A).

Functional marker aldh7 was designed as the CAPS primer. The forward and reverse primer sequences for aldh7 were 5′-GGCTACTTTCTAGGTGGTTG-3′ and 5′-ACTCTTTGGTCCTGTTCTTT-3′, respectively. Restriction enzyme *SmII* was used for the digestion of the amplicons. The size of the PCR amplicons without the *SmII* cutting site was 287 bp, and with the *SmII* cutting site, the amplicon sizes were 89 and 198 bp, respectively (Figure 3B).

Another functional marker ge was designed as a dCAPS primer by using dCAPS Finder 2.0 [44]. The forward and reverse primer sequences for ge were 5′-GGGACGGACACGGTGGCGATCTTTA-3′ and 5′-GTCTCCTTCACGATGGACT-3′, respectively. The restriction enzyme *MseI* was used for the digestion of the amplicons. The PCR amplicon size was 176 bp without the *MseI* cutting site, and the sizes were 24 and 152 bp with the cutting site (Figure 3C).

Polymorphic markers for background selection were chosen from Gramene (http://www.gramene.org/markers/microsat/ (accessed on 1 May 2014), RGP (http://rgp.dna.affrc.go.jp/ (accessed on 1 May 2014), and studies by Hsu et al. [45] and Shen et al. [46]. All markers were tested on the parental lines of the PFR and RFR populations to validate the polymorphism. For the PFR population, 101 markers were screened in the F_2_ generation (Figure S1, Table S2). For the RFR population, 35 and 37 markers were tested on the BC_1_F_1_ and BC_2_F_1_ populations, respectively. Ninety-one markers were tested in the BC_2_F_2_ population (Figure S2, Table S3).

### 4.3. DNA Isolation and PCR Amplification

Genomic DNA was extracted from young leaf tissues, using the benzyl chloride protocol [47]. The isolated DNA was diluted to 20 ng/µL for polymerase chain reaction (PCR); the reaction mixture (10 µL) contained 20 ng genomic DNA, 0.2 µM (each) forward and reverse primers, 5 µL Multiplex PCR Master Mix (QIAGEN, Inc., Valencia, CA, USA), and 1 µL Q-Solution (QIAGEN, Inc., Valencia, CA, USA). PCR was performed using a thermocycler (GeneAmp PCR System 9700, PerkinElmer Corp., Norwalk, CT, USA) as per the following conditions: initial denaturation at 94 °C for 2 min; 30 cycles of amplification at 94 °C for 30 s, 55 °C for 20 s, and 72 °C for 30 s; and a final extension at 72 °C for 2 min.

Restriction digestion of the amplicons was performed using the *SmII* restriction enzyme; the reaction mixture was composed of 5 µL PCR product, 1 µL 10× Cutsmart, 0.5 µL restriction enzyme, and 3.5 µL ddH_2_O. The mixture was incubated at 55 °C for 3 h. For *MseI*, the reaction volume was composed of 5 µL PCR product, 1 µL 10× NEBuffer 4, 0.5 µL restriction enzyme, and 3.5 µL ddH_2_O. The mixture was incubated at 37 °C for 3 h.

The PCR products after restriction digestion were separated on 6% non-denatured polyacrylamide gel electrophoresis (PAGE) in 0.5× TBE at 100 V (Dual Triple-Wide Mini-Vertical System, C. B. S. Scientific, San Diego CA, USA) for 60 min. The gels were stained with SYBR Safe DNA gel stain (Life Technologies) and visualized under UV light (365 nm).

### 4.4. Antioxidant Analysis

#### 4.4.1. Total Phenolic Content

Total phenolic content was measured as described by Bao et al. [48], with slight modifications. The extract (20 μL) was diluted with methanol (180 μL). Samples and standard solutions prepared with gallic acid were mixed with 0.5 mL 0.5 N Folin–Ciocalteu reagent, and the reaction was neutralized with 7.5% sodium carbonate (1 mL). After incubation for 30 min, absorbance at 760 nm was measured using a spectrometer. A calibration curve was prepared using gallic acid solution. The total phenolic content was expressed as milligrams of gallic acid equivalent (mg GAE) per gram of dry weight (mg GAE g^−1^).

#### 4.4.2. Flavonoid Contents

Flavonoid content was measured as described by Bao et al. [48], with slight modifications. The extract (100 μL) was diluted with methanol (100 μL). Samples and standard solutions prepared with rutin were mixed with 0.15 mL 5% sodium nitrite, 0.15 mL 10% AlCl_3_·6H_2_O, and 1 mL NaOH (1M). After incubation for 30 min, the absorbance was measured at 415 nm using a spectrometer. A calibration curve was constructed using rutin. The total flavonoid content was expressed as milligrams of rutin equivalent per gram of dry weight (mg RE g^−1^).

#### 4.4.3. DPPH Scavenging Activity (%)

DPPH activity was measured as described by Hsieh et al. [49], with slight modifications. The extract (100 μL) was diluted with methanol (1.9 mL). Samples and standard solutions were mixed with 1.5 mL 0.008% DPPH. After incubation for 30 min, the absorbance was measured at 517 nm using a spectrometer. Methanol was used as a blank. Scavenging activity (%) = (A517 nm blank–A517 sample)/(A517 nm blank) × 100.

#### 4.4.4. Monomeric Anthocyanin Pigment Content

Anthocyanin content was measured as described by Lee et al. [50], with slight modifications. The samples were divided into two groups and subjected to a different type of treatment. The extract (75 μL) in one group was mixed with 1.8 mL 0.025M KCL (pH = 1.0), and in the other group it was mixed with 1.8 mL 0.4M sodium acetate (pH = 4.5). After incubation for 30 min, the absorbance was measured at 520 and 700 nm. The anthocyanin pigment concentration was expressed as cyanidin-3-glucoside equivalents (mg/L) = (A × MW × DF × 1000) ÷ (ε × l). A = (A520 nm–A700 nm) pH = 1.0–(A520 nm–A700 nm) pH = 4.5; DF = dilution factor; MW = molecular weight for cyanidin-3-glucoside = 449.2 g/mol; ε= molar absorptivity for cyaniding-3-glucoside = 26,900 L cm^−1^ mol^−1^; l = pathlength in cm.

### 4.5. Statistical Analysis

Data were analyzed using the R software (version 4.0.5). Analysis of variance (ANOVA) was performed, and the least significant difference (LSD) method was used for multiple comparisons. The Graphical Geno Types (GGT) software (Version 2.0) was used to estimate the recurrent parent genome (RPG) recovery rate based on marker data [51].

## 5. Conclusions

The breeding procedure for both PFR32 and RFR13 lasted for 10 years and reached the breeding goals by combing giant embryos and golden-like traits in colored rice. It proves that for the biofortification purpose, the combination of molecular markers and traditional breeding methods can create a strategy for functional food development. PFR32 and RFR13 can become valuable products and adapt to the current agricultural community.

## Figures and Tables

**Table 1:** Five-season yield trials of the purple functional rice population (PFR) and red functional rice population (RFR).

Lines	2017-1		2017-2		2018-1		2018-2		2019-1	
CNY922401	7377.0	abcd	5088.4	e	8532.5	a	1422.1	abc	8221.4	ab
PFR4	6354.9	cdef	-		-		-		-	
PFR5	6510.5	bcdef	4355.1	f	-		-		-	
PFR20	6043.8	ef	-		-		-		-	
PFR24	5955.0	f	-		-		-		-	
PFR32	7599.2	ab	5066.2	e	7599.2	ab	911.0	c	7999.2	b
TNGSW26	7910.3	a	6621.6	a	6666.0	ab	1177.7	bc	9065.8	a
RFR1	7732.6	ab	-		-		-		-	
RFR2	7221.5	abcde	5466.1	cde	-		-		-	
RFR13	7510.4	abc	5466.1	cde	7799.2	ab	2733.1	ab	7799.2	b
RFR16	6354.9	cdef	5732.8	cd	-		-		-	
RFR21	5755.0	f	-		-		-		-	
RFR22	6577.1	bcdef	-		-		-		-	
RFR34	6754.9	abcdef	-		-		-		-	
RFR36	6754.9	abcdef	5288.4	de	6043.8	b	2955.3	a	-	
RFR39	6132.7	def	-		-		-		-	
RFR44	6621.6	bcdef	6199.4	ab	6377.1	b	1133.2	c	-	
RFR46	6488.2	bcdef	-		-		-		-	
RFR48	6354.9	cdef	-		-		-		-	
RFR51	7621.5	ab	5910.5	bc	6821.5	ab	1799.8	abc	6710.4	c

Least significant difference (LSD) method was used for multiple comparisons. Different lowercase letters indicate different groups (*p* < 0.05).

**Table 2:** DPPH scavenging activity, and anthocyanin content of the 20 lines, including recurrent parents, CNY922401, and TNGSW26 in 2017.

Line	DPPH (%) ^x^		Anthocyanin Content ^y^	
CNY922401	88.89	ab	1.48	d
PFR4	87.58	abc	2.25	a
PFR5	86.32	bc	2.09	abc
PFR20	86.83	abc	2.15	ab
PFR24	85.52	bc	1.84	bc
PFR32	87.63	abc	1.81	c
TNGSW26	82.4	bc	nd	
RFR1	85.72	bc	nd	
RFR2	87.63	abc	nd	
RFR13	81.25	c	nd	
RFR16	37.31	g	nd	
RFR21	27.9	h	nd	
RFR22	27.45	h	nd	e
RFR34	86.07	bc	0.11	e
RFR36	93.06	a	0.22	e
RFR39	88.24	ab	0.34	e
RFR44	86.17	bc	0.05	
RFR46	67.17	e	nd	
RFR48	50.18	f	nd	
RFR51	74.16	d	nd	

^x^: values are expressed as DPPH scavenging activity (%); ^y^: values are expressed as cyanidin-3-glucoside mg g^−1^ DW. Least significant difference (LSD) method was used for multiple comparisons. Means with different letters in the same column are not significantly different at the 5% level. Different lowercase letters indicate different groups (*p* < 0.05). nd indicates “not detected”.

**Table 3:** Total phenolic content, flavonoid content, DPPH scavenging activity, and anthocyanin content of the seven lines, including recurrent parents, CNY922401, and TNGSW26 in 2018.

Line	Total Phenolic Content ^w^		Flavonoid Content ^x^		DPPH (%) ^y^		Anthocyanin Content ^z^	
CNY922401	5.41	a	1.94	a	84.36	a	2.34	a
PFR32	4.51	a	1.59	b	75.73	ab	1.66	b
TNGSW26	0.73	c	0.89	c	58.98	c	0.03	d
RFR13	2.44	b	0.85	c	64.00	bc	0.08	d
RFR36	1.62	bc	0.81	c	45.46	d	0.19	c
RFR44	0.70	c	0.59	d	36.44	d	0.04	d
RFR51	2.08	b	0.82	c	70.35	bc	0.05	d

^w^: Values are expressed as gallic acid mg g^−1^ DW; ^x^: Values are expressed as rutin mg g^−1^ DW; ^y^ Values are expressed as DPPH scavenging activity (%); ^z^ Values are expressed as cyanidin-3-glucoside mg g^−1^ DW. Least significant difference (LSD) method was used for multiple comparisons. Means with different letters in the same column are not significantly different at the 5% level. Different lowercase letters indicate different groups (*p* < 0.05).

**Table 4:** Total phenolic content, flavonoid content, DPPH scavenging activity, and anthocyanin content of CNY922401, PFR32, TNGSW 26, RFR13 and Taiken 9 (TK9).

Line	Total Phenolic Content ^w^		Flavonoid Content ^x^		DPPH (%) ^y^		Anthocyanin Content ^z^	
CNY922401	4.58	a	1.38	a	84.79	b	1.64	a
PFR32	2.37	bc	1.40	a	86.30	ab	1.06	b
TNGSW26	3.55	ab	0.82	b	90.59	a	0.06	c
RFR13	1.58	c	0.63	c	88.63	ab	0.03	c
TK9	1.15	c	0.20	d	34.47	c	0.01	c

^w^: Values are expressed as gallic acid mg g^−1^ DW; ^x^: Values are expressed as rutin mg g^−1^ DW; ^y^ Values are expressed as DPPH scavenging activity (%); ^z^ Values are expressed as cyanidin-3-glucoside mg g^−1^ DW. Least significant difference (LSD) method was used for multiple comparisons. Means with different letters in the same column are not significantly different at the 5% level. Different lowercase letters indicate different groups (*p* < 0.05).

## Supplementary Materials

The following supporting information can be downloaded at: https://www.mdpi.com/article/10.3390/ijms23094678/s1.
