# Supplementary material for: The Development of Two High-Yield and High-Quality Functional Rice Cultivars Using Marker-Assisted Selection and Conventional Breeding Methods
Source: Int J Mol Sci. 2022 Apr 23;23(9):4678. doi: 10.3390/ijms23094678 (PMC9102896; doi:10.3390/ijms23094678)
Supplement: Supplementary file 1 [file ijms-23-04678-s001.zip › Figure S1.pdf]

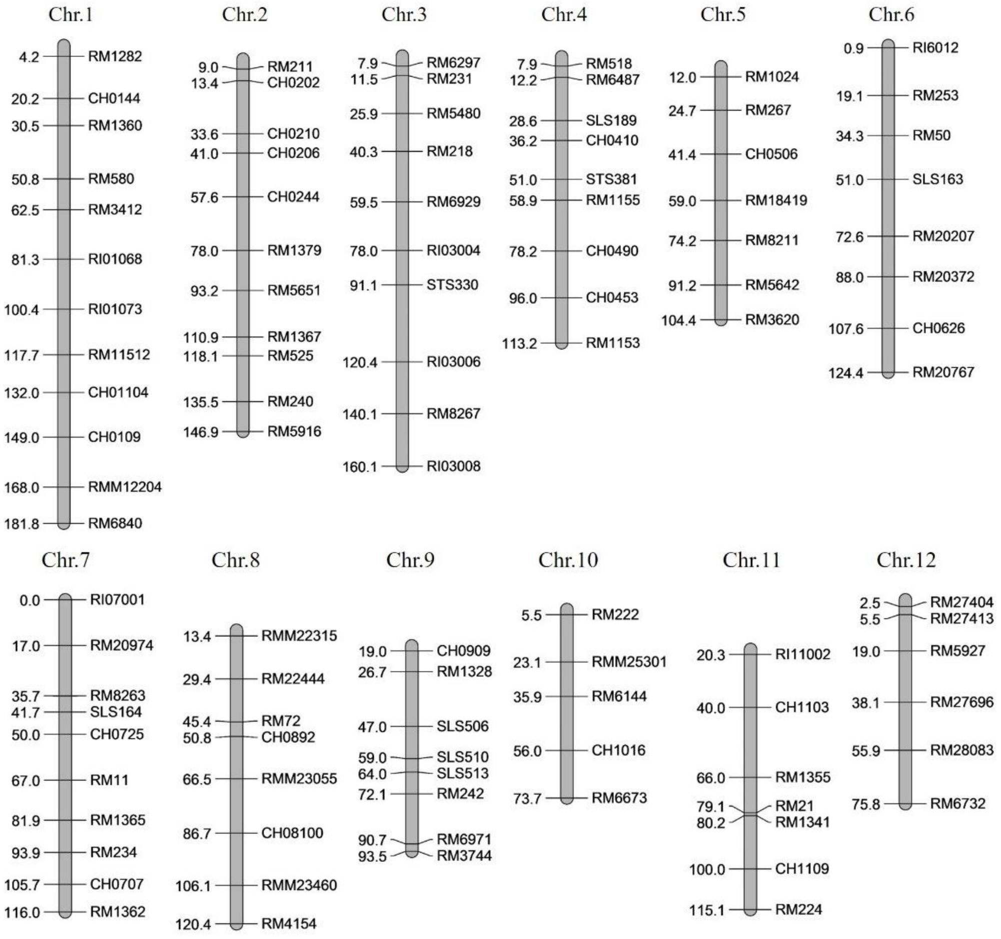

Figure S1. The polymorphic markers used for background selection of the purple functional rice population and the distribution of polymorphic markers on each chromosome. A total of 101 markers were used for background selection in the F_2 generation.
