# Supplementary material for: The Development of Two High-Yield and High-Quality Functional Rice Cultivars Using Marker-Assisted Selection and Conventional Breeding Methods
Source: Int J Mol Sci. 2022 Apr 23;23(9):4678. doi: 10.3390/ijms23094678 (PMC9102896; doi:10.3390/ijms23094678)
Supplement: Supplementary file 1 [file ijms-23-04678-s001.zip › Figure S2.pdf]

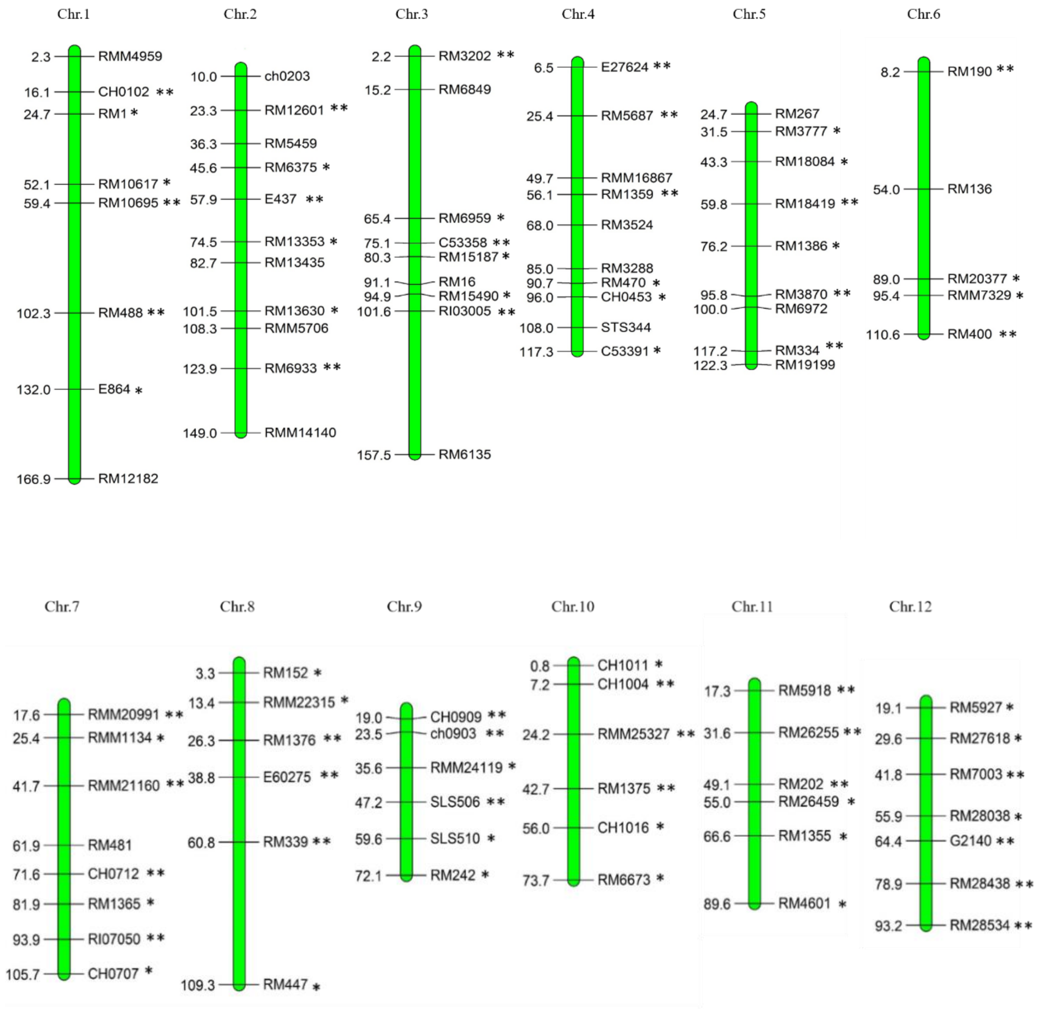

Figure S2. The polymorphic markers used for background selection of the red functional rice populations and the distribution of polymorphic markers on each chromosome. Thirty-five markers (labelled with one asterisk) were used for background selection in the BC_1F_1 generation, 37 markers (labelled with double asterisks) were used in the BC_2F_1 generation, and 91 markers were used in th

e BC_2F_2 generation.
